# Vascular Risk Factors and Diseases Modulate Deficits of Reward-Based Reversal Learning in Acute Basal Ganglia Stroke

**DOI:** 10.1371/journal.pone.0155267

**Published:** 2016-05-10

**Authors:** Ulla K. Seidel, Janine Gronewold, Manon Wicking, Christian Bellebaum, Dirk M. Hermann

**Affiliations:** 1 Department of Neurology, University Hospital Essen, University of Duisburg-Essen, Germany; 2 Institute of Experimental Psychology, Heinrich Heine University, Düsseldorf, Germany; Julius-Maximilians-Universität Würzburg, GERMANY

## Abstract

**Background:**

Besides motor function, the basal ganglia have been implicated in feedback learning. In patients with chronic basal ganglia infarcts, deficits in reward-based reversal learning have previously been described.

**Methods:**

We re-examined the acquisition and reversal of stimulus-stimulus-reward associations and acquired equivalence in eleven patients with acute basal ganglia stroke (8 men, 3 women; 57.8±13.3 years), whose performance was compared eleven healthy subjects of comparable age, sex distribution and education, who were recruited outside the hospital. Eleven hospitalized patients with a similar vascular risk profile as the stroke patients but without stroke history served as clinical control group.

**Results:**

In a neuropsychological assessment 7±3 days post-stroke, verbal and spatial short-term and working memory and inhibition control did not differ between groups. Compared with healthy subjects, control patients with vascular risk factors exhibited significantly reduced performance in the reversal phase (F[2,30] = 3.47; p = 0.044; post-hoc comparison between risk factor controls and healthy controls: p = 0.030), but not the acquisition phase (F[2,30] = 1.01; p = 0.376) and the acquired equivalence (F[2,30] = 1.04; p = 0.367) tasks. In all tasks, the performance of vascular risk factor patients closely resembled that of basal ganglia stroke patients. Correlation studies revealed a significant association of the number of vascular risk factors with reversal learning (r = -0.33, p = 0.012), but not acquisition learning (r = -0.20, p = 0.121) or acquired equivalence (r = -0.22, p = 0.096).

**Conclusions:**

The previously reported impairment of reward-based learning may be attributed to vascular risk factors and associated diseases, which are enriched in stroke patients. This study emphasizes the necessity of appropriate control subjects in cognition studies.

## Introduction

The basal ganglia are extensively connected to neocortical and limbic structures.[1] Initially, the basal ganglia were implicated in motor control.[2] Later studies found that the basal ganglia are involved in various cognitive functions including inhibition control,[3] reward processing,[4] decision making,[5] procedural memory,[6] and habit and skill learning.[7]

Observations of implicit learning deficits in Parkinson´s and Huntington´s patients[6, 8, 9] prompted Bellebaum et al.[10] to study reward-based learning in stroke patients, who, based on the defined nature of their brain injury, may provide insights into brain-behavior relationships. In comparison to healthy control subjects, reward-based reversal learning in a probabilistic feedback task was selectively impaired in patients with chronic basal ganglia stroke, suggesting that patients may exhibit deficits in feedback learning. For the clinical neurologist, feedback learning is highly relevant for rehabilitation success, since it likely affects stroke recovery processes.

A problem of studies in degenerative disorders like Parkinson’s or Huntington’s disease is that neurodegeneration not exclusively affects the basal ganglia. In the earlier stroke study,[10] reward-based learning was assessed in a heterogeneous patient group, in which the time since stroke was highly variable ranging from 16 to 101 months and which included patients with incidentally detected clinically silent infarcts. Since these patients were not studied in the acute stroke phase, associated factors other than the stroke may have influenced test results. Patients suffering from stroke usually present with vascular risk profiles that in addition to stroke influence cognitive function.[11]

In the study by Bellebaum et al. [10], the consequences of the stroke and those of vascular risk factors could not be disentangled, since basal ganglia stroke patients were compared with healthy control subjects that lacked vascular risk factors. To discriminate the differential impact of the stroke and associated risk factors, we re-evaluated deficits of reward-based learning in patients with acute first-ever basal ganglia stroke, which we compared with two different control groups of the same age, sex and education, one of which comprised patients suffering from vascular risk factors without stroke history. For reasons of data comparability, we used the identical probabilistic feedback learning paradigm, as reported before [10].

## Material and Methods

### Participants

Eleven consecutive patients with acute clinical first-ever basal ganglia stroke recruited via the stroke unit at the University hospital Essen during their hospital stay received a standardized interview to collect demographic data and a medical history including information about known cardiovascular risk factors, and associated illnesses (specifically coronary artery disease including myocardial infarct [CAD], left ventricular insufficiency, cardiac rhythm abnormalities, stroke/transient ischemic attack, and peripheral artery disease [PAD]). Age ≥55 years, arterial hypertension (defined by systolic blood pressure ≥ 140 mmHg, diastolic blood pressure ≥ 90 mmHg or treatment with antihypertensive drugs), diabetes (defined by physician-diagnosed diabetes, blood glucose levels ≥200 mg/dl, fasting glucose levels ≥126 mg/dl, or treatment with antidiabetic medication), hypercholesterolemia (defined by LDL≥160 mg/dl, HDL≤40 mg/dl or treatment with cholesterol-lowering drugs), and current smoking were noted, overweight classified as body-mass index >25 kg/m^2^. In addition to a physical examination, a 1.5 T brain MRI including T2-weighted, T2*-weighted and diffusion-weighted sequences was performed in all except two patients. In one of the two patients, which both received a computed tomography scan, MRI could not be performed because of a cardiac pacemaker, in the other the primary brain hemorrhage could unequivocally be detected on computed tomography grounds. For the stroke patients, two separate control groups matched for age and sex and with comparable education were recruited. The first group was recruited via University notice boards (called 'healthy control subjects' in the following). The second group was recruited at the Department of Cardiology of the University hospital and consisted of hospitalized patients with a vascular risk profile comparable to the stroke patients but without stroke history (called 'risk factor patients'). The study was approved by the ethical committee of the University Duisburg-Essen, all subjects gave written informed consent.

### Assessment of control variables

To control for more general deficits that might influence their performance in reward-based learning, all participants underwent a battery of five standardized tests, namely the Wechsler Adult Intelligence Scale (subtests similarities and picture completion; assessment of verbal comprehension and perceptual reasoning),[12] the Wechsler Revised Memory Scale (subtests digit and block span forward and backward; assessment of verbal and spatial short-term and working memory)[13] and the Go/Nogo task of the Test for Attentional Performance (assessment of inhibition control).[14] Clinically relevant aphasia, neglect and apraxia were excluded with the Token Test (subtest of Aachen Aphasia Test),[15] Cats test[16] and standardized meaningful voluntary movements, respectively.

### Assessment of reward-based learning

**Fig 1:**
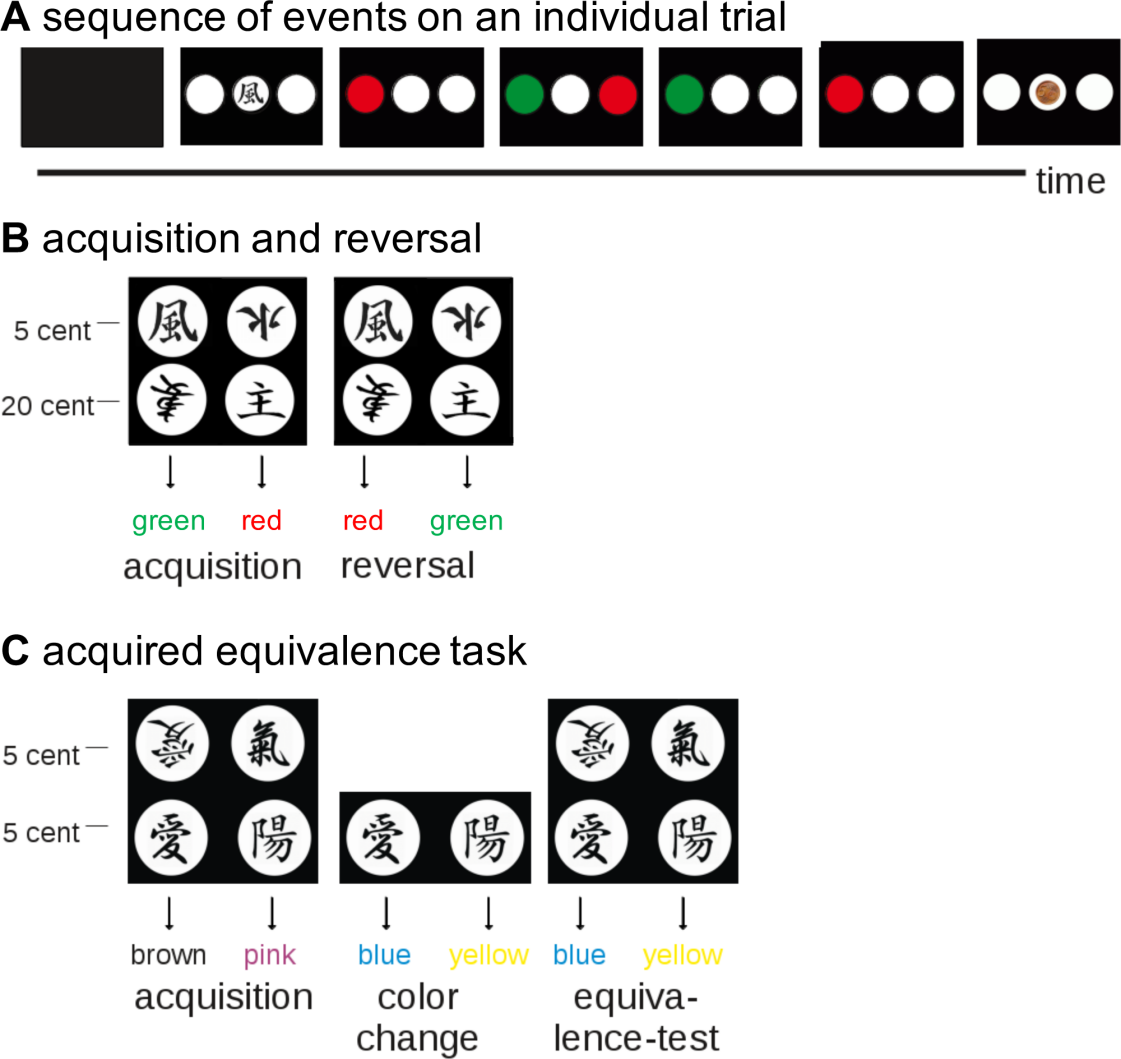
Reward-based learning paradigm. (A) After the presentation of an Asian symbol, subjects were asked to select one of the two colours by pressing the corresponding button. The decision was followed by outcome presentation (reward or non-reward). (B) In the first learning task, subjects were asked to learn associations between symbols and colours, followed by a reversal of contingencies. (C) In the acquired equivalence task, subjects had to relearn associations after a colour change and were then expected to transfer the newly learned associations to previously presented symbols (modified from [10]).

Probabilistic learning tasks mainly involve non-declarative learning processes because of non-deterministic stimulus feedback associations.[17] As described by Bellebaum et al.,[10] reward-based learning in the present study was assessed in two probabilistic learning tasks the first of which included an acquisition and a reversal phase and the second consisted of an acquired equivalence task, in which Asian symbols had to be associated with colours based on reward feedback (Fig 1A). Stimuli were presented with Presentation 12.2 (Neurobehavioral Systems Inc.; http://www.neuro-bs.com). The data were analyzed with Matlab 7.7.0.

#### Learning task 1: Reward-based acquisition and reversal

In the acquisition phase participants had to learn associations between four abstract Asian symbols and two colours (red, green) with the help of monetary feedback (Fig 1B). Two symbols were associated with red, two symbols with green. For two symbols (one associated with red, the other with green), correct choices were associated with a 20-cent reward and for the other two symbols with a 5-cent reward. On each trial, one of the four symbols was shown on the screen for 8 s, followed by two coloured circles (red and green), in left and right positions on a monitor, which were shown for up to 8 s. During presentation of these circles, subjects were asked to select one of the two colours by pressing a left or right response button. The choice either led to a monetary reward (indicated by a coin in a white circle in the middle of the screen) or to non-reward, indicated by three empty white circles. Unknown to the subjects, each of the four symbols was probabilistically associated with red or green: For each symbol, the choice of one colour led to reward in 80% of the cases and to non-reward in 20% of the cases. This probabilistic relationship between choices and outcomes made sure that subjects used non-declarative learning strategies. The choice of the other colour was never rewarded. The acquisition phase comprised 120 trials, divided into three blocks of 40 trials, i.e. each symbol was presented 30 times, 10 times per block. For each block, the locations of the red and green circles relative to the centre (left or right) were counterbalanced. Similarly, the symbol-colour associations were counterbalanced across subjects. In the reversal phase, participants had to learn that the symbol-colour associations were reversed: Symbols initially associated with red were now associated with green and vice versa (Fig 1B). As acquisition, reversal consisted of three blocks of 40 trials each.

#### Learning task 2: Reward-based acquired equivalence

The probabilistic acquired equivalence task started with two acquisition phases (acquisition and colour change) and was followed by a cognitive transfer phase (equivalence test). Participants again had to learn associations between four Asian symbols and two colours. Each symbol was again associated with one colour, and two symbols were associated with the same colour. A correct choice led to reward in 80% of the trials, whereas the alternative choice was never rewarded. The structure of an individual trial was identical to the first learning task; reward magnitude was, however, kept constant (5 cents), and new symbols and colours (brown and pink) were used (Fig 1C). During initial acquisition, participants had to learn that two symbols were associated with the colour pink and the other two symbols were associated with the colour brown. Acquisition was completed when the subject had reached a criterion of eight correct responses in a row (minimum of 38 trials; maximum of 80 trials). In the second acquisition phase (colour change), new colours were presented (blue and yellow) and only two of the four symbols (one initially associated with pink, the other with brown) were used. One of the symbols was now associated with yellow, the other one with blue. In the transfer phase, the newly learned associations between the new colours and the symbols had to be transferred to the symbols used during initial learning, which requires participants to recognize that two symbols are equivalent in the sense that they are always associated with the same colour (Fig 1C). The transfer phase started, when subjects had correctly responded five times in a row in the second acquisition phase (minimum of 15 trials, maximum of 80 trials). The transfer phase consisted of 40 trials presented in one block and there was no feedback on individual trials. Subjects were informed about their cumulative winnings every five trials.

### Statistical analysis

Continuous data are expressed as mean ± standard deviation unless otherwise specified. Age and education were compared between the three groups (stroke patients, risk factor patients and healthy controls) by one-way ANOVA using the between-subjects factor group. Sex and presence of vascular risk factors were compared by chi-square tests or Fisher exact tests. For the reward-based acquisition and reversal phase, a mixed design ANOVA with the between-subjects factor group (stroke patients, healthy control subjects and risk factor patients), the within-subjects factor block (1 to 3) and the within-subjects factor reward magnitude (RM; 5 and 20 cent) was calculated with the number of correct responses as dependent variable. In case of significant main effects, Fisher's least significant difference (LSD) post-hoc tests were calculated.

For the two acquisition phases of the reward-based acquired equivalence, the number of patients reaching the learning criterion (8 successive correct responses in phase 1, 5 successive correct responses in phase 2) was compared between groups by chi-square tests. For the equivalence test phase, ANOVA with the between-subjects factor group and the within-subjects factor symbol type (symbols used to learn the new color-symbol associations in the second acquisition phase vs transfer symbols) was calculated because stimuli were presented in one block now and reward magnitude was kept constant. Correlations were calculated using Kendall´s tau-b statistic. Data were evaluated using SPSS 21.0 for Windows. Single case analyses were done with the freeware singlims, a software that is designed to analyze small sample sizes with modified t-tests.[18] P values <0.05 were considered significant.

## Results

### Patient characteristics

The demographics and risk factor profile of stroke patients, risk factor patients and healthy controls as well as the localization of infarcts in stroke patients are summarized in Table 1. Of eleven stroke patients (8 men, 3 women), ten had an ischemic stroke and one a primary brain hemorrhage. The mean age of stroke patients was 57.8±13.3 years. All ischemic infarcts were territorial infarcts (diameter >10 mm). In six ischemic stroke patients, the stroke had presumable macroangiopathic etiology. In 4 ischemic stroke patients, the stroke had presumable cardioembolic etiology. The brain hemorrhage in one patient had hypertensive etiology. None of the stroke patients had previous silent strokes, none had cerebral microbleeds. Three stroke patients had no white matter lesions, three slight white matter lesions, four moderate white matter lesions and one severe white matter lesions. Slight brain atrophy was found in two of eleven stroke patients. The mean time-point of neuropsychological assessment after the stroke was 7±3 days. Risk factors and associated vascular diseases did not differ between stroke patients and control patients with vascular risk factors without stroke (all p>0.198). Except for overweight and smoking, healthy control subjects revealed no vascular risk factors.

**Table 1:** Characteristics of the healthy control-, risk factor-, and stroke patients: Sociodemographics, risk factors and performance in reward-based learning as well as infarct localization for the stroke patients.

	Age (years)	Sex	Education (years)	Infarct hemisphere	Infarct localisation	Vascular risk factors	Acquisition	Reversal	Equivalence test
**Healthy control subject 1**	45	Male	13			Overweight	105†	108†	34†
**Healthy control subject 2**	74	Male	13				93†	93	35†
**Healthy control subject 3**	52	Male	10			Smoking, overweight	58	78	27
**Healthy control subject 4**	64	Male	8			Overweight	53	71	24
**Healthy control subject 5**	64	Male	10			Overweight	66	87	18
**Healthy control subject 6**	79	Female	9				55	65	24
**Healthy control subject 7**	41	Male	10			Smoking, overweight	77	66	16
**Healthy control subject 8**	49	Female	13				96†	110†	31
**Healthy control subject 9**	51	Female	10			Overweight	93†	110†	20
**Healthy control subject 10**	62	Male	10				71	91	35†
**Healthy control subject 11**	47	Male	9				59	60	20
**Risk factor patient 1**	44	Male	8			Arterial hypertension, diabetes, overweight	60	61	20
**Risk factor patient 2**	74	Male	8			Arterial hypertension	64	51	18
**Risk factor patient 3**	53	Male	8			PAD, arterial hypertension, hypercholesterolemia, diabetes	55	65	22
**Risk factor patient 4**	69	Male	8			CAD, overweight	49	57	20
**Risk factor patient 5**	66	Male	13			CAD, arterial hypertension, smoking	71	91	27
**Risk factor patient 6**	86	Female	10			Arterial hypertension	69	83	26
**Risk factor patient 7**	42	Male	10			CAD	77	56	31
**Risk factor patient 8**	44	Female	10			Arterial hypertension, smoking	75	82	24
**Risk factor patient 9**	53	Female	9			Mild left ventricular insufficiency	55	64	27
**Risk factor patient 10**	64	Male	8			Overweight	77	57	26
**Risk factor patient 11**	52	Male	13			CAD, diabetes, hypercholesterolemia, overweight	60	59	23
**Stroke Patient 1**	44	Male	10	Left	Caudal putamen	Hypercholesterolemia, smoking	69	54	24
**Stroke Patient 2**	75	Male	8	Left	Caudal putamen	CAD, diabetes, arterial hypertension, overweight	65	51	21
**Stroke Patient 3**	53	Male	10	Left	Dorsal putamen	Diabetes, arterial hypertension, overweight	68	48	18
**Stroke Patient 4**	69	Male	8	Left	Caudal putamen	Arterial hypertension	54	55	17
**Stroke Patient 5**	66	Male	10	Left	Multiple lesions rostral putamen and pallidum	Atrial fibrillation, diabetes, arterial hypertension	61	67	25
**Stroke Patient 6**	83	Female	8	Left	Caudate head and rostral putamen	Sick sinus syndrome (pacemaker), arterial hypertension, hypercholesterolemia	26*†	31*†	29
**Stroke Patient 7**	43	Male	9	Left	Dorsal putamen	Arterial hypertension, hypercholesterolemia, smoking	76	105†	36†
**Stroke Patient 8**	51	Female	13	Left	Caudate head and rostral putamen	Atrial fibrillation	103	103†	17
**Stroke Patient 9**	50	Female	10	Right	Caudal putamen	Arterial hypertension, smoking, overweight	101	37*	14†
**Stroke Patient 10**	53	Male	9	Left	Dorsal putamen (primary hemorrhage)	Overweight	94	91	27
**Stroke Patient 11**	49	Male	9	Left	Dorsal putamen	Arterial hypertension, hypercholesterolemia, smoking, overweight	80	83	15†

Data were analyzed by single case comparisons.

*p<0.05 compared with healthy control subjects

†p<0.05 compared with risk factor patients.

**Fig 2:**
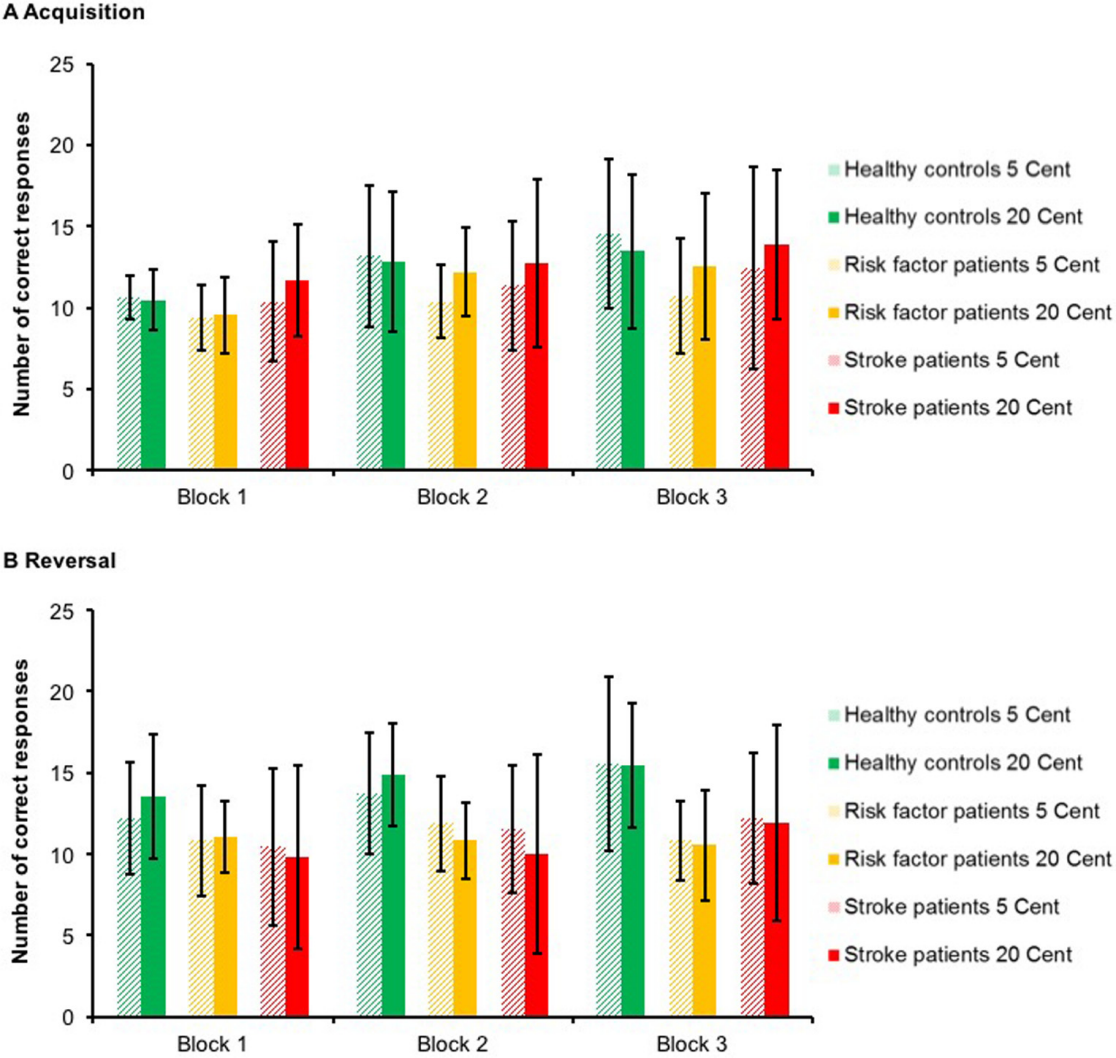
Performance of healthy control subjects, control patients without stroke with vascular risk factors (‘risk factor patients’) and stroke patients in the reward-based acquisition and reversal, broken down into learning blocks and reward magnitude. Data are means of correct responses with S.D. values. ANOVA revealed a significant main effect for the factor group in the reversal phase [F(2,30) = 3.47; p = 0.044] reflecting a significantly lower number of correct responses in risk factor patients than healthy controls (p = 0.032). The performance of stroke patients and risk factor patients was very similar (p = 0.999).

Mean values acquisition: 75.1±18.8; 64.7±9.7 and 72.5±22.4 in healthy control subjects, risk factor patients and stroke patients, respectively (see also Fig 2).

Mean values reversal: 85.4±18.7; 66.0±13.2 and 65.9±25.9 in healthy control subjects, risk factor patients and stroke patients, respectively (see also Fig 2).

Mean values equivalence test: 25.8±7.0; 24.0±3.9 and 22.1±6.8 in healthy control subjects, risk factor patients and stroke patients, respectively (see also Fig 2).

### Reward-based acquisition and reversal

For the acquisition phase, ANOVA with the factors group (stroke patients, healthy control subjects and risk factor patients), block (1 to 3) and reward magnitude (RM; 5 and 20 cent) yielded a significant main effect of block [F(1.33,39.77) = 11.32; p = 0.001] with more correct responses in the later stages of the experiment (block 1 vs block 2: p = 0.007; block 1 vs block 3: p = 0.003; block 2 vs block 3: p = 0.092) (Fig 2). Besides, no significant main or interaction effects were found (all p>0.076).

For the reversal phase, a significant main effect of group [F(2,30) = 3.47; p = 0.044] reflected a significantly lower number of correct responses in stroke patients and risk factor patients compared to healthy controls (p = 0.029 and 0.030, respectively). The performance of stroke patients and risk factor patients was very similar (p = 0.992). Additionally, a significant block effect [F(2,60) = 4.32; p = 0.018] with more correct responses in block 3 compared to block 1 was noticed (p = 0.048). Besides, no significant main or interaction effects were identified (all p>0.142). Excluding the patient with primary hemorrhage led to almost identical results; stroke patients and vascular risk factor patients again performed significantly worse than controls (data not shown).

Since the performance in the reversal phase is dependent on the performance of the acquisition phase - in order to learn that associations between the Asian symbols and the colours have changed (reversal), participants first had to learn how the Asian symbols were paired with colours (acquisition)—, we additionally analyzed difference values between reversal and acquisition by subtracting the number of correct responses at the end of the acquisition phase (block 3) from the number of correct responses in the reversal phase for each block, symbol and reward magnitude. Here, no significant main or interaction effects emerged (all p>0.060). For the group factor, a trend was observed (p = 0.077), which was based on a trend for stroke patients scoring lower than healthy controls.

To evaluate possible effects related to defined stroke localizations, single case comparisons were performed, in which the total number of correct responses of individual patients in block 1-3 were compared with both control groups (Table 1). There was no association between lesion location and acquisition and reversal performance. The only stroke patient exhibiting significant deficits in reversal learning as compared to both control groups was much older than all other patients and exhibited a severe cardiac disease related to sick sinus syndrome.

### Reward-based acquired equivalence

Performance in the three acquired equivalence phases did not differ between groups. Only four of the eleven stroke patients reached both learning criteria (compared to five risk factor patients and six healthy controls, p = 0.565). In the equivalence test, i.e., the last phase of the reward-based acquired equivalence task, ANOVA with the factors group and symbol type yielded a significant effect of symbol type [F(1,30) = 8,41; p = 0.007] with more correct responses for symbols used to learn the new color-symbol associations in the second acquisition phase compared to transfer symbols. No additional group or interaction effects were detected (all p>0.194). In single case comparisons, no striking significance patterns were found (Table 1).

### Association of cardiovascular risk factors with reward-based learning

**Fig 3:**
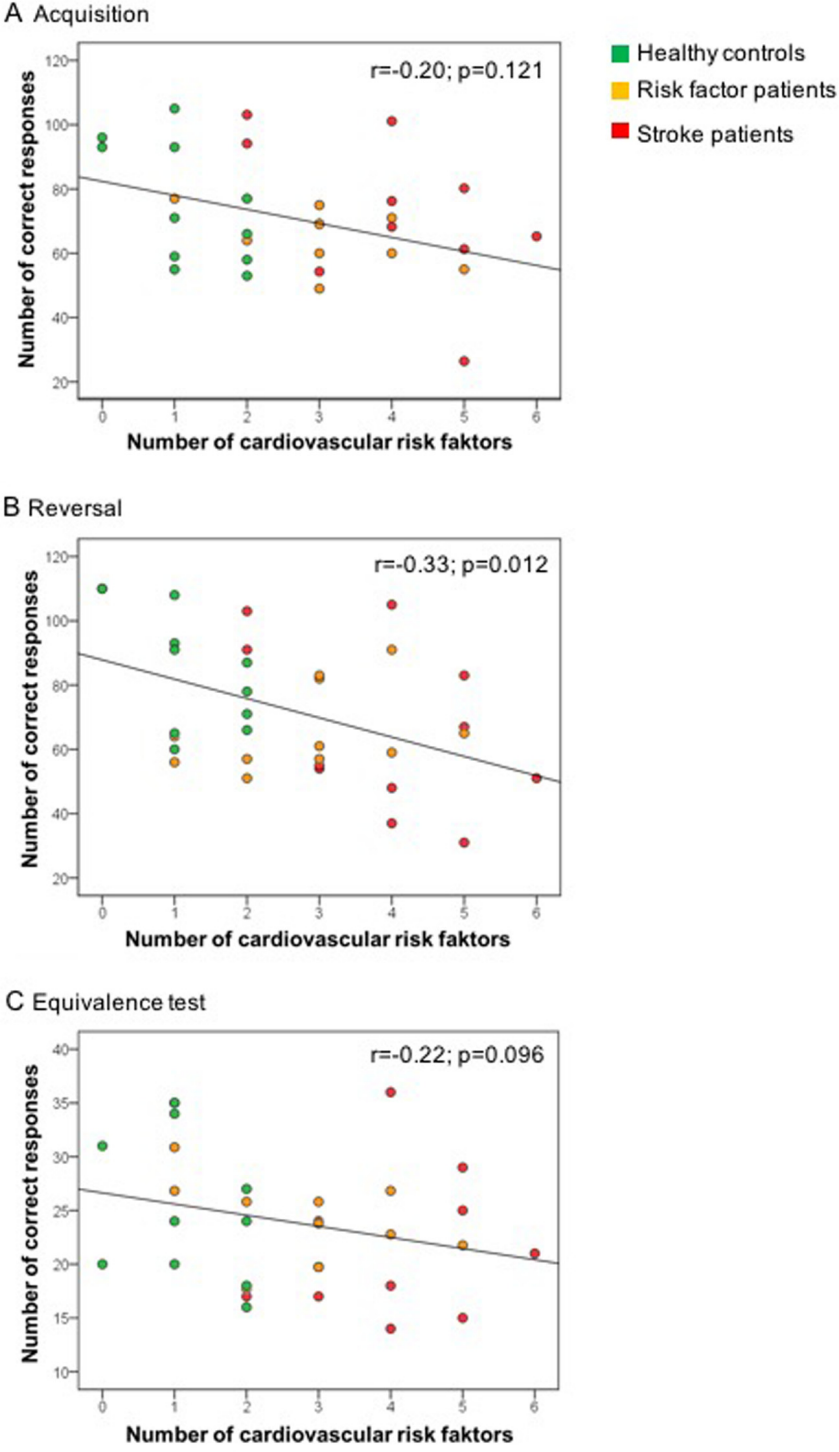
Correlation between the number of vascular risk factors and the number of correct responses in the reward-based acquisition and reversal and equivalence test. Data were analyzed by Kendall’s tau-b correlations. Note that there was a significant correlation between the number of risk factors and correct responses in the reversal, but no significant correlation in the acquisition phase and equivalence test.

Since reversal learning differed between healthy control subjects and risk factor patients, the participants' performance in each of the learning phases was correlated with the number of risk factors, as presented in Table 1 and S1 Table. This analysis revealed a significant correlation between the number of risk factors and correct responses in the reward-based reversal (r = -0.33; p = 0.012), but no significant correlation in the acquisition phase (r = -0.20; p = 0.121) and equivalence test (r = -0.22; p = 0.096) for the total study cohort (Fig 3). To further define the impact of vascular risk factors on the performance in reward-based learning, we calculated multivariable linear regressions including the number of vascular risk factors, age, sex and education for the whole cohort of 33 subjects. These analyses confirmed an association between the number of vascular risk factors and the number of correct responses in the reward-based reversal (B = -4.49, 95% CI = -8.51 to 0.14; p = 0.057) but not the acquisition (B = -1.77, 95% CI = -5.13 to 1.59; p = 0.289) and acquired equivalence test (B = -1.05, 95% CI = -2.49 to 0.40; p = 0.149) phase.

### Working memory and inhibition control

Because working memory and inhibition control are important requirements for reward-based learning, we evaluated whether the participants' working memory and inhibition control differed between groups and whether the performance in each of the learning tasks was correlated with working memory and inhibition control. Working memory and inhibition control did not differ between healthy control subjects, risk factor patients and stroke patients (all p>0.071). Furthermore, reversal learning did not correlate with working memory or inhibition control (S1 Table). Since we observed a significant correlation between risk factors and reversal learning, and risk factors could also influence performance in reversal learning indirectly by impairing memory and inhibition control, we finally calculated partial correlations between number of vascular risk factors and number of correct responses in the reversal phase adjusting for measures of working and short term memory and inhibition control. After adjustment, the significance of the association between the number of risk factors and the number of correct responses on the reversal phased did not decrease (p = 0.050 adjusted for digit span forward; p = 0.007 for digit span backward; p = 0.013 for block span forward; p = 0.021 for block span backward; p = 0.008 for inhibition control), supporting the validity of an independent influence of risk factors on reversal learning deficits.

## Discussion

By examining reward-based learning in a group of eleven patients with acute basal ganglia stroke, whose performance was compared to two different control groups of comparable age, sex and education, one of which had a very similar vascular risk profile as the stroke patients, we showed that the previously reported deficit in reward-based reversal learning in basal ganglia stroke patients is modulated by vascular risk factors and associated diseases. Basal ganglia stroke patients had very similar reward-based learning performance as control patients with risk factors without stroke. Across the total study cohort, reversal learning significantly correlated with the number of vascular risk factors.

The observation that patients with basal ganglia stroke have deficits in reward-based reversal learning has previously been reported in a patient sample of the same size (11 patients) that was compared with healthy control subjects matched for age and intellectual ability.[10] This more heterogeneous cohort included patients with chronic stroke with a wide range of time periods from stroke until study participation and clinically silent infarcts in two patients that had incidentally been detected. A control group of subjects suffering from vascular risk factors comparable to those of stroke patients was lacking. Cognitive deficits in vascular risk patients are widely considered to be attributed to cerebral microangiopathy, which is a consequence of long-term risk factor exposure.[11] However, risk factors, e.g. arterial hypertension and dyslipidemia, have also been shown to predict cognitive impairment independent of cerebral microangiopathy,[19–21] possibly by disturbing cerebral autoregulation. As in this study, stroke patients did not reveal deficits in acquisition learning, acquired equivalence, short-term and working memory, and inhibition control. These data suggest that reversal learning is particularly sensitive to vascular risk factors and diseases.

Deficits in reward-based acquisition and reversal learning have repeatedly been described in Parkinson´s and Huntington´s disease.[6, 8, 9] Similar to stroke patients, Parkinson´s patients are typically old and not rarely present vascular risk factors. Parkinson´s patients exhibit more fundamental deficits in implicit learning and memory, reward processing, habit and skill learning.[3, 6, 7, 9] Neurodegeneration in Parkinson´s disease is usually bilateral and not primarily affects the basal ganglia, but substantia nigra and brain stem.[22] Compared with Parkinson's patients, Huntington´s patients are young. Huntington's patients show severe recall and recognition memory impairments that not rarely fulfill criteria of dementia.[23] The putamen, more specifically its GABAergic spiny neurons, is bilaterally affected by the neurodegenerative process, which in addition involves the substantia nigra and also cortex.[24]

Using a well-defined sample of patients with acute basal ganglia stroke, this study does not provide evidence that acute basal ganglia injury induces deficits in reward-based learning. When acquisition performance was taken into account in analyzing reversal learning, a trend towards reduced performance did, however, emerge for the stroke patients. In any case, this study suggests that the vascular risk profile is important for reward-based learning. Although having the same patient number and the same testing protocol as Bellebaum et al.,[10] the observations of this study are limited by the small sample size, which offered limited possibilities for adjustments in regression analyses. As another shortcoming, our control groups did not undergo cerebral imaging so that we could not adjust statistical models for cerebral microangiopathy which could have influenced cognitive performance. The present results emphasize the importance of appropriate control groups in the study of stroke-related cognitive deficits. Based on our findings, the role of the basal ganglia in reward-based learning further needs to be scrutinized.

## Supporting Information

S1 Table Correlations between verbal and spatial short-term memory, verbal and spatial working memory and inhibition control with reward-based learning. Data were analyzed by Kendall’s tau-b correlations. Note the absence of correlation of verbal working memory, spatial working memory and inhibition control with reward-based reversal learning.
(DOCX)
