# Supplementary material for: Vascular Risk Factors and Diseases Modulate Deficits of Reward-Based Reversal Learning in Acute Basal Ganglia Stroke
Source: PLoS One. 2016 May 10;11(5):e0155267. doi: 10.1371/journal.pone.0155267 (PMC4862682; doi:10.1371/journal.pone.0155267)
Supplement: S1 Table — Data were analyzed by Kendall’s tau-b correlations. Note the absence of correlation of verbal working memory, spatial working memory and inhibition control with reward-based reversal learning. (DOCX) [file pone.0155267.s001.docx]

**Supporting Information**

**S1 Table.** Correlations between verbal and spatial short-term memory, verbal and spatial working memory and inhibition control with reward-based learning

|  | **Acquisition** | | **Reversal** | | **Equivalence test** | |
| --- | --- | --- | --- | --- | --- | --- |
| **Healthy control subjects** | **r** | **p** | **r** | **p** | **r** | **p** |
| Verbal short-term memory | 0.25 | 0.646 | 0.35 | 0.298 | 0.26 | 0.439 |
| Spatial short-term memory | 0.43 | 0.188 | 0.26 | 0.443 | 0.43 | 0.188 |
| Verbal working memory | 0.24 | 0.482 | 0.24 | 0.470 | 0.15 | 0.666 |
| Spatial working memory | 0.26 | 0.439 | 0.26 | 0.440 | 0.35 | 0.298 |
| Inhibition control | -0.05 | 0.883 | -0.22 | 0.520 | -0.51 | 0.113 |
| **Risk factors patients** |  | | | | | |
| Verbal short-term memory | 0.50 | 0.119 | 0.33 | 0.326 | 0.44 | 0.179 |
| Spatial short-term memory | -0.04 | 0.905 | 0.26 | 0.434 | -0.21 | 0.527 |
| Verbal working memory | **0.68** | **0.021** | 0.05 | 0.895 | **0.74** | **0.009** |
| Spatial working memory | 0.12 | 0.723 | -0.32 | 0.345 | 0.03 | 0.931 |
| Inhibition control | **-0.72** | **0.013** | -0.32 | 0.343 | -0.58 | 0.059 |
| **Stroke patients** |  | | | | | |
| Verbal short-term memory | 0.52 | 0.104 | 0.33 | 0.315 | -0.07 | 0.841 |
| Spatial short-term memory | 0.43 | 0.191 | 0.41 | 0.213 | -0.09 | 0.792 |
| Verbal working memory | -0.19 | 0.587 | -0.43 | 0.192 | 0.17 | 0.618 |
| Spatial working memory | **0.63** | **0.037** | 0.48 | 0.139 | -0.13 | 0.699 |
| Inhibition control | -0.28 | 0.433 | -0.37 | 0.291 | -0.24 | 0.500 |

Data were analyzed by Kendall’s tau-b correlations. Note the absence of correlation of verbal working memory, spatial working memory and inhibition control with reward-based reversal learning.
